# Electrophilic Trifluoromethylselenolation of Boronic Acids

**DOI:** 10.3390/molecules22050833

**Published:** 2017-05-19

**Authors:** Clément Ghiazza, Anis Tlili, Thierry Billard

**Affiliations:** 1 Institute of Chemistry and Biochemistry (ICBMS-UMR CNRS 5246), Université de Lyon, Université Lyon 1, CNRS, F-69622 Lyon, France; clementghiazza@gmail.com (C.G.); anis.tlili@univ-lyon1.fr (A.T.); 2 CERMEP—In Vivo Imaging, Groupement Hospitalier Est, F-69677 Lyon, France

**Keywords:** trifluoromethylselenolation, boronic acids, trifluoromethylselenyl chloride, fluorine, selenium

## Abstract

Trifluoromethylselenylated compounds are emergent compounds with interesting physicochemical properties that still suffer from a lack of efficient synthetic methods. We recently developed an efficient one-pot strategy to generate in situ CF_3_SeCl and use it in various reactions. Herein, we continue our study of the reactivity scope of this preformed reagent. Cross-coupling reactions with aromatic and heteroaromatic boronic acids have been investigated. The expected products have been obtained, using a stoichiometric amount of copper, with moderate yields.

## 1. Introduction

Fluorinated compounds play a more and more important role in various fields of application. Among all the fluorinated substituents, the CF_3_ group occupies a particular place due to its specific properties [1,2,3,4,5,6,7,8,9,10,11,12,13]. Furthermore, the association of this CF_3_ moiety with chalcogens leads to new fluorinated substituents with very interesting electronic and physicochemical properties. This has been well illustrated by CF_3_O- and CF_3_S-molecules [14,15,16,17,18], especially due to their high lipophilicities (Hansch–Leo parameters: π_R_(OCF_3_) = 1.04, π_R_(SCF_3_) = 1.44) [19] which contribute to their favoring of membrane permeation and, consequently, increase their bioavailability. In the series of chalcogens, the CF_3_Se group has been less investigated. However, selenylated compounds also play an important role in various fields of application, from materials to life sciences [20,21,22,23,24,25,26,27,28,29,30,31]. This is illustrated, for example, by the drug Ebselen [32,33,34,35]. Furthermore, the Hansch–Leo lipophilicity parameter of the CF_3_Se group has been recently measured to π_R_(SeCF_3_) = 1.29 [36].

Despite the strong interest in this group, synthetic methods to obtain trifluoromethylselenylated molecules are still limited. Trifluoromethylation of selenylated compounds has recently been investigated for the first time [37,38,39,40,41,42,43,44,45,46,47,48,49]. Although this strategy gave good results, the preliminary preparation of selenylated adducts can be a drawback. Late stage direct trifluoromethylselenolation appears to be the most versatile approach. Nucleophilic reactions have been the most investigated, with a large panel of organic compounds from nucleophilic substitutions onto halogen compounds or diazonium salts, to cross-coupling reactions with halogen substrates or boronic acids [50,51,52,53,54,55,56,57,58,59,60,61,62,63,64]. Nevertheless, this approach required the preparation of CF_3_Se^−^ anions using a stoichiometric amount of selenium metal. Electrophilic trifluoromethylselenolations have been less described. The only available reagent for such reactions is CF_3_SeCl, which is volatile, potentially toxic and, until recently, difficult to synthesize [65,66].

To favor a safe and easy handling of this reagent, we have recently described an efficient procedure to generate in situ this species from benzyl trifluoromethyl selenide (**1**). This strategy has already been applied to electrophilic aromatic substitutions [67] and reactions with Grignard reagents and lithium alkynides [36].

## 2. Results and Discussion

In our objective to extend the scope of the reactivity of CF_3_SeCl, following our one-pot strategy, we decided to study the trifluoromethylselenolation of boronic acids.

The reaction was first optimized with biphenyl boronic acid (**2a**). All the attempts are summarized in Table 1.

**Figure 1:**
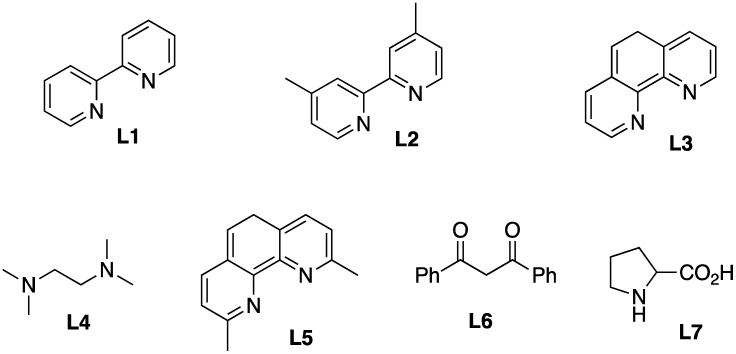
Ligands used in Table 1.

The use of CuI as a catalyst with bipyridine **L1** led to the expected compounds with a very low yield (Entry 1). With Cu(OAc)_2_, a better yield was observed, but was still low (Entry 2). In order to improve this encouraging result, the base or ligand first had to be removed. Without the base, the reaction failed, although a small amount of **3a** was observed without **L1** (Entries 3–4). This led us to reduce the quantity of the ligand, and 40% of **3a** was then formed (Entry 5). Next, various other ligands (Figure 1) were screened without success (Entries 6–12); bipyridine **L1** remained the more efficient.

The influence of the nature of the base was then explored. A good yield was obtained with Cs_2_CO_3_, whereas K_3_PO_4_ led to a similar result to that of K_3_CO_3_ (Entries 13–14). Surprisingly, CsF, often used in cross-coupling reactions with boronic acid, provided a low yield (Entry 15). Organic nitrogen bases appeared to be deleterious for the reaction (Entries 16–17). This could be explained by a competitive copper coordination between these bases and **L1**. At higher temperatures, no improvement was observed but, on contrary, this resulted in a decrease of yield (Entry 18). This may be due to the outgassing of the highly volatile CF_3_SeCl reagent. 

Catalytic amounts of copper (II) and ligand were then tested, but lower yields were observed (Entries 19–20). Again, heating proved to be deleterious (Entry 21). Inspired by our previous work with a sulfur series [68], some water (7 eq.) was added resulting, in this case, in a non-significant effect (Entry 22). Consequently, stoichiometric conditions (Entry 13) remained the better ones.

**Scheme 1:**
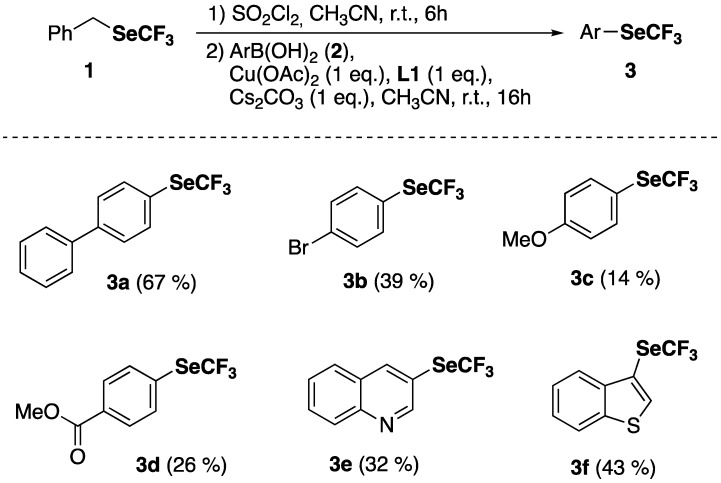
Trifluoromethylselenolation of aromatic boronic acids. Yields shown are those of the isolated products.

These conditions were applied to other aromatic boronic acids (Scheme 1).

Only moderate yields were obtained with substituted aromatic compounds, whatever the donor or acceptor electronic character of the substituents. In heteroaromatic series, the same moderate results were observed.

When these lukewarm results were obtained, some amounts of CF_3_SeSeCF_3_ were detected as well as homocoupling products from the boronic reagents. This could be rationalized by the high reactivity of CF_3_SeCl, which leads to a competition between the kinetically low coupling reaction and the more rapid dimerization. The homocoupling reaction could then come from the lack of CF_3_SeCl for the expected reaction. Despite the use of an excess of preformed CF_3_SeCl, no better results were observed. Furthermore, during the preliminary formation of CF_3_SeCl, one equivalent of benzyl chloride was also formed, which could possibly disturb the cross-coupling reaction. 

## 3. Materials and Methods

Commercial reagents were used as supplied. Reagent **1** was synthesized following procedures described in the literature [36,67]. Anhydrous solvents were used as supplied. NMR spectra were recorded on a Bruker AV 400 (Billerica, MA, USA) spectrometer at 400 MHz (^1^H-NMR), 101 MHz (^13^C-NMR), and 376 MHz (^19^F-NMR), or on a Bruker AV 300 spectrometer at 300 MHz (^1^H-NMR) and 282 MHz (^19^F-NMR). Multiplicities are indicated as follows: s (singlet), d (doublet), t (triplet), q (quartet), p (quintet), sext (sextet), m (multiplet), b (broad). All coupling constants are reported in Hz.

### 3.1. Synthesis of Benzyl Trifluoromethyl Selenide *(**1**)*

To a dry round-bottom flask equipped with a magnetic stirrer, benzylselanocyanate (13.7 g, 70.0 mmol, 1.0 equiv.) and dry THF (140 mL) were added. The flask was evacuated and refilled with nitrogen three times, and then trifluoromethyl trimethylsilane (TMSCF_3_) (20.7 mL, 140 mmol, 2.0 equiv.) was added. The reaction mixture was cooled to 0 °C, and then tetrabutylammonium fluoride (TBAF) in THF 1 M (14.0 mL, 14.0 mmol, 0.2 equiv.) was added dropwise. After 10 min at 0 °C under nitrogen, the reaction was allowed to warm to 23 °C and was stirred for 7 h. The conversion was checked by ^19^F-NMR with PhOCF_3_ as an internal standard. The reaction mixture was then partitioned between water and pentane, and the aqueous layer was extracted with pentane. The combined organic layers were washed with brine, dried over MgSO_4_, filtered through a pad of silica (rinsed with pentane) and concentrated to dryness (under moderate vacuum). The crude residue was purified by chromatography (pentane: 100) to afford the desired product **1** as a colorless liquid (11.7 g, 70% yield). ^1^H-NMR (300 MHz, CDCl_3_): δ = 7.37−7.27 (massif, 5H), 4.26 (s, 2H). ^19^F-NMR (282 MHz, CDCl_3_): δ = −34.47 (s, 3F). The results are in accordance with the literature [38].

### 3.2. Typical Procedure

*Solution A*: To a flame-dried flask equipped with a magnetic stirrer, BnSeCF_3_ (**1**) (0.40 mmol, 1.1 equiv.), SO_2_Cl_2_ (0.40 mmol, 1.1 equiv.) and anhydrous acetonitrile (1 mL) were added under nitrogen. The reaction mixture was stirred for 6 h at 20 °C. 

*Solution B*: To a flame-dried flask equipped with a magnetic stirrer, biphenylboronic acid **2** (0.36 mmol, 1 equiv.), copper (II) acetate (0.36 mmol, 1 equiv.), bypirydine (**L1**) (0.36 mmol, 1 equiv.) and cesium carbonate (0.36 mmol, 1 equiv.) were added under nitrogen. 

Solution A was then poured into solution B by syringe and the mixture was stirred at 20 °C for 16 h. Conversion was checked by ^19^F-NMR with PhOCF_3_ as an internal standard. The reaction mixture was partitioned between CH_2_Cl_2_ and water. The aqueous layer was extracted with CH_2_Cl_2_ and the combined organic layers were washed with brine, dried over MgSO_4_, filtered and concentrated to dryness. The crude residue was purified by flash chromatography to afford the desired product **3**. 
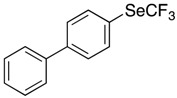


*Synthesis of 4-[(trifluoromethyl)selanyl]-1,1′-biphenyl* (**3a**). Eluent for flash chromatography: cyclohexane/AcOEt 98:2. ^1^H-NMR (300 MHz, CDCl_3_) δ = 7.83 (m, 2H), 7.65−7.60 (massif, 4H), 7.50 (m, 2H), 7.42 (m, 1H). ^19^F-NMR (282 MHz, CDCl_3_) δ = −36.05 (s, 3F). The results are in accordance with the literature [57]. 
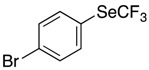


*Synthesis of 1-bromo-4-[(trifluoromethyl)selanyl]benzene* (**3b**). Eluent for flash chromatography: pentane 100%. ^1^H-NMR (300 MHz, CDCl_3_) δ = 7.60 (m, 2H), 7.53 (m, 2H). ^19^F-NMR (282 MHz, CDCl_3_) δ = −36.03 (s, 3F). The results are in accordance with the literature [61]. 
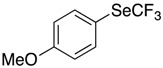


*Synthesis of 1-methoxy-4-[(trifluoromethyl)selanyl]benzene* (**3c**). Eluent for flash chromatography: cyclohexane/toluene 9:1. ^1^H-NMR (300 MHz, CDCl_3_) δ = 7.66 (m, 2H), 6.91 (m, 2H), 3.83 (s, 3H). ^19^F-NMR (282 MHz, CDCl_3_) δ = −37.18 (s, 3F). The results are in accordance with the literature [61]. 
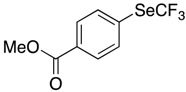


*Synthesis of methyl 4-[(trifluoromethyl)selanyl]benzoate* (**3d**). Eluent for flash chromatography: cyclohexane/EtOAc 97:3 to 95:5. ^1^H-NMR (300 MHz, CDCl_3_) δ = 8.04 (m, 2H), 7.81 (m, 2H), 3.94 (s, 3H). ^19^F-NMR (282 MHz, CDCl_3_) δ = −35.21 (s, 3F). The results are in accordance with the literature [57]. 
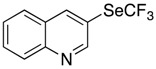


*Synthesis of 3-[(trifluoromethyl)selanyl]quinoline* (**3e**). Eluent for flash chromatography: cyclohexane/Et_2_O 8:2. ^1^H-NMR (300 MHz, CDCl_3_) δ = 9.14 (s, 1H), 8.65 (d, *J* = 1.8 Hz, 1H), 8.21 (d, *J* = 8.7 Hz, 1H), 7.86 (m, 2H), 7.67 (m, 1H). ^19^F-NMR (282 MHz, CDCl_3_) δ = −35.50 (s, 3F). The results are in accordance with the literature [53]. 
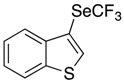


*Synthesis of 3-[(trifluoromethyl)selanyl]-1-benzothiophene* (**3f**). Eluent for flash chromatography: cyclohexane/toluene 98:2. ^1^H-NMR (300 MHz, CDCl_3_) δ = 8.02 (d, *J* = 7.7 Hz, 1H), 7.96 (s, 1H), 7.92 (m, 1H), 7.51 (m, 1H), 7.44 (m, 1H). ^19^F-NMR (282 MHz, CDCl_3_) δ = −35.66 (s, 3F). The results are in accordance with the literature [57].

## 4. Conclusions

In our study of its reactivity scope, we have demonstrated that CF_3_SeCl, in situ preformed, could react with boronic acids to perform trifluoromethylselenolation of aromatic or heteroaromatic compounds. However, moderate yields were generally observed due to the overly high reactivity of CF_3_SeCl and the presence of generated benzyl chloride. This points out the major issue of this one-pot strategy; the subsequently formed benzyl chloride may limit this approach by inducing side-reactions. Furthermore, the high reactivity of CF_3_SeCl, which can easily dimerize, could also constitute a drawback with reactions which are kinetically too low. This underlines the necessity of developing new reagents, that are isolable, easy to handle and have a modular reactivity that is easier to control.

## Figures and Tables

**Table 1:**
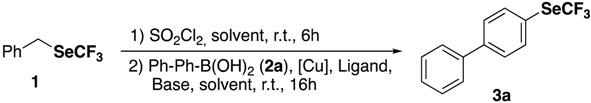
Coupling reaction between CF_3_SeCl, generated in situ, and boronic acid **3a**.

Entry	[Cu]	Ligand ^a^	Base	Solvent	3a (%) ^b^
1	CuI (1 eq.)	**L1** (2 eq.)	K_2_CO_3_ (1 eq.)	CH_3_CN	5
2	Cu(OAc)_2_ (1 eq.)	**L1** (2 eq.)	K_2_CO_3_ (1 eq.)	CH_3_CN	23
3	Cu(OAc)_2_ (1 eq.)	**L1** (2 eq.)	-	CH_3_CN	0
4	Cu(OAc)_2_ (1 eq.)	-	K_2_CO_3_ (1 eq.)	CH_3_CN	4
5	Cu(OAc)_2_ (1 eq.)	**L1** (1 eq.)	K_2_CO_3_ (1 eq.)	CH_3_CN	40
6	Cu(OAc)_2_ (1 eq.)	**L2** (1 eq.)	K_2_CO_3_ (1 eq.)	CH_3_CN	14
7	Cu(OAc)_2_ (1 eq.)	**L3** (1 eq.)	K_2_CO_3_ (1 eq.)	CH_3_CN	3
8	Cu(OAc)_2_ (1 eq.)	**PPh_3_** (1 eq.)	K_2_CO_3_ (1 eq.)	CH_3_CN	0
9	Cu(OAc)_2_ (1 eq.)	**L4** (1 eq.)	K_2_CO_3_ (1 eq.)	CH_3_CN	6
10	Cu(OAc)_2_ (1 eq.)	**L5** (1 eq.)	K_2_CO_3_ (1 eq.)	CH_3_CN	3
11	Cu(OAc)_2_ (1 eq.)	**L6** (1 eq.)	K_2_CO_3_ (1 eq.)	CH_3_CN	0
12	Cu(OAc)_2_ (1 eq.)	**L7** (1 eq.)	K_2_CO_3_ (1 eq.)	CH_3_CN	0
13	Cu(OAc)_2_ (1 eq.)	**L1** (1 eq.)	Cs_2_CO_3_ (1 eq.)	CH_3_CN	70
14	Cu(OAc)_2_ (1 eq.)	**L1** (1 eq.)	K_3_PO_4_ (1 eq.)	CH_3_CN	48
15	Cu(OAc)_2_ (1 eq.)	**L1** (1 eq.)	CsF (1 eq.)	CH_3_CN	6
16	Cu(OAc)_2_ (1 eq.)	**L1** (1 eq.)	Et_3_N (1 eq.)	CH_3_CN	0
17	Cu(OAc)_2_ (1 eq.)	**L1** (1 eq.)	Pyridine (1 eq.)	CH_3_CN	0
18 ^c^	Cu(OAc)_2_ (1 eq.)	**L1** (1 eq.)	Cs_2_CO_3_ (1 eq.)	CH_3_CN	37
19	Cu(OAc)_2_ (0.2 eq.)	**L1** (0.2 eq.)	Cs_2_CO_3_ (1 eq.)	CH_3_CN	56
20	Cu(OAc)_2_ (0.2 eq.)	**L1** (0.4 eq.)	Cs_2_CO_3_ (1 eq.)	CH_3_CN	50
21 ^c^	Cu(OAc)_2_ (0.2 eq.)	**L1** (0.2 eq.)	Cs_2_CO_3_ (1 eq.)	CH_3_CN	-
22 ^d^	Cu(OAc)_2_ (0.2 eq.)	**L1** (0.2 eq.)	Cs_2_CO_3_ (1 eq.)	CH_3_CN	60

^a^ See Figure 1 for ligand structures. ^b^ Yield was determined by ^19^F-NMR spectroscopy by using PhOCF_3_ as an internal standard. ^c^ 50 °C instead of r.t. ^d^ Addition of 7 eq. of H_2_O.
